# Risk of thyroid as a first or second primary cancer. A population‐based study in Italy, 1998–2012

**DOI:** 10.1002/cam4.4193

**Published:** 2021-09-17

**Authors:** Emanuele Crocetti, Veronica Mattioli, Carlotta Buzzoni, Silvia Franceschi, Diego Serraino, Salvatore Vaccarella, Stefano Ferretti, Susanna Busco, Ugo Fedeli, Massimo Varvarà, Fabio Falcini, Manuel Zorzi, Giuliano Carrozzi, Walter Mazzucco, Cinzia Gasparotti, Silvia Iacovacci, Federica Toffolutti, Rossella Cavallo, Fabrizio Stracci, Antonio G. Russo, Adele Caldarella, Stefano Rosso, Antonino Musolino, Lucia Mangone, Claudia Casella, Mario Fusco, Giovanna Tagliabue, Daniela Piras, Rosario Tumino, Linda Guarda, Ylenia M. Dinaro, Silvano Piffer, Pasquala Pinna, Guido Mazzoleni, Anna C. Fanetti, Luigino Dal Maso, for AIRTUM working group

**Affiliations:** ^1^ Cancer Epidemiology Unit Centro di Riferimento Oncologico di Aviano (CRO) IRCCS Aviano Italy; ^2^ Global Patient Outcomes and Real World Evidence (GPORWE) International Eli Lilly Italy S.p.A Sesto Fiorentino Florence Italy; ^3^ AIRTUM Database (in charge until January 2019) Florence Italy; ^4^ Section of Cancer Surveillance International Agency for Research on Cancer Lyon France; ^5^ Romagna Cancer Registry Section of Ferrara Local Health Unit University of Ferrara Ferrara Italy; ^6^ Cancer Registry of Latina Province ASL Latina Latina Italy; ^7^ Epidemiological Department Azienda Zero Padua Italy; ^8^ Registro Tumori Integrato Catania‐Messina‐Siracusa‐Enna Università degli Studi di Catania Catania Italy; ^9^ Romagna Cancer Registry Istituto Scientifico Romagnolo per lo Studio e la Cura dei Tumori (IRST) IRCCS Meldola Italy; ^10^ Veneto Tumor Registry Azienda Zero Padua Italy; ^11^ Modena Cancer Registry Public Health Department AUSL Modena Modena Italy; ^12^ Palermo and Province Cancer Registry Clinical Epidemiology Unit with Cancer Registry Azienda Ospedaliera Universitaria Policlinico “Paolo Giaccone” University of Palermo Palermo Italy; ^13^ Brescia Cancer Registry Epidemiology Unit Brescia Health Protection Agency Brescia Italy; ^14^ ASL Salerno Cancer Registry Salerno Italy; ^15^ Public Health Section Department of Medicine and Surgery University of Perugia Perugia Italy; ^16^ Cancer Registry of Milan Epidemiology Unit Agency for Health Protection Milan Italy; ^17^ Tuscany Cancer Registry Clinical Epidemiology Unit Institute for Cancer Research, Prevention and Clinical Network (ISPRO) Florence Italy; ^18^ Piedmont Cancer Registry Azienda Ospedaliera‐Universitaria Città della Salute e della Scienza di Torino Italy; ^19^ Parma Cancer Registry Oncology Unit Azienda Ospedaliera Universitaria di Parma Parma Italy; ^20^ Reggio Emilia Cancer Registry Epidemiology Unit AUSL ASMN‐IRCCS Azienda USL di Reggio Emilia Reggio Emilia Italy; ^21^ Liguria Cancer Registry Clinical Epidemiology IRCCS Ospedale Policlinico San Martino Genova Italy; ^22^ Cancer Registry of ASL Napoli 3 Sud Napoli Italy; ^23^ Lombardy Cancer Registry Cancer Registry Unit Department of Research Fondazione IRCCS Istituto Nazionale dei Tumori Milan Italy; ^24^ North Sardinia Cancer Registry Azienda Regionale per la Tutela della Salute Sassari Italy; ^25^ Cancer Registry and Histopathology Department Provincial Health Authority (ASP 7) Ragusa Italy; ^26^ Mantova Cancer Registry Epidemilogy Unit Agenzia di Tutela della Salute (ATS) della Val Padana Mantova Italy; ^27^ Siracusa Cancer Registry Health Unit of Siracusa Siracusa Italy; ^28^ Trento Province Cancer Registry Unit of Clinical Epidemiology Trento Italy; ^29^ Nuoro Cancer Registry RT Nuoro ASSL Nuoro/ATS Sardegna Nuoro Italy; ^30^ Southtyrol Cancer Registry Bolzano Italy; ^31^ Sondrio Cancer Registry Health Protection Agency Sondrio Italy

**Keywords:** cancer survivors, Italy, population‐based cancer registries, relative risk, second primary cancer, thyroid cancer

## Abstract

**Background:**

The number of patients living after a cancer diagnosis is increasing, especially after thyroid cancer (TC). This study aims at evaluating both the risk of a second primary cancer (SPC) in TC patients and the risk of TC as a SPC.

**Methods:**

We analyzed two population‐based cohorts of individuals with TC or other neoplasms diagnosed between 1998 and 2012, in 28 Italian areas covered by population‐based cancer registries. Standardized incidence ratios (SIRs) of SPC were stratified by sex, age, and time since first cancer.

**Results:**

A total of 38,535 TC patients and 1,329,624 patients with other primary cancers were included. The overall SIR was 1.16 (95% CI: 1.12–1.21) for SPC in TC patients, though no increase was shown for people with follicular (1.06) and medullary (0.95) TC. SPC with significantly increased SIRs was bone/soft tissue (2.0), breast (1.2), prostate (1.4), kidney (2.2), and hemolymphopoietic (1.4) cancers. The overall SIR for TC as a SPC was 1.49 (95% CI: 1.42–1.55), similar for all TC subtypes, and it was significantly increased for people diagnosed with head and neck (2.1), colon–rectum (1.4), lung (1.8), melanoma (2.0), bone/soft tissue (2.8), breast (1.3), corpus uteri (1.4), prostate (1.5), kidney (3.2), central nervous system (2.3), and hemolymphopoietic (1.8) cancers.

**Conclusions:**

The increased risk of TC after many other neoplasms and of few SPC after TC questions the best way to follow‐up cancer patients, avoiding overdiagnosis and overtreatment for TC and, possibly, for other malignancies.

## Graphical abstract

This is the first study able to calculate population‐based risk of thyroid cancers as a first or second tumor separately for different thyroid cancer histological types (i.e., follicular, medullary, and poorly differentiated). In a context of a large proportion of thyroid cancer cases due to overdiagnosis, the findings of the present study may help to more focused primary prevention and surveillance for side effects of treatments, thus avoiding overtreatment, particularly among younger women.

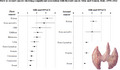

## 1 INTRODUCTION

The number of patients living after a cancer diagnosis is rising in Italy,^1^ as well as in several other countries.^2^ In particular, one of the largest 10‐year increases is foreseen for thyroid cancer (TC) patients (+79% between 2010 and 2020),^1^ largely due to the impact of overdiagnosis on the rapidly increasing TC incidence,^3^, ^4^ the modest improvements of survival,^5^ and substantially stable mortality rates.^3^ The vast majority of people living after a TC diagnosis have the same life expectancy as the general population (i.e., they are *cured*),^6^ in particular those who were overdiagnosed.^7^ Notably, overdiagnosis of asymptomatic TC accounted for 75% of cases in Italian women and 63% in Italian men, between 1998 and 2012.^3^

In Italy, more than 3.4 million people are living after a cancer diagnosis other than TC.^1^ They may be at risk of developing a new primary cancer due to several reasons, including common etiologic factors (i.e., environmental exposures, genetics, and lifestyles), late effects of cancer treatments, and, possibly, enhanced surveillance.^8^, ^9^ In addition, several studies from Europe and the United States have reported a substantial risk of TC detection during other cancers follow‐up.^10^, ^11^, ^12^, ^13^ A higher than expected incidence rate of second primary cancer (SPC) in TC patients has been reported and recently confirmed.^13^, ^14^ In particular, an increased incidence has been consistently reported for breast cancer,^15^, ^16^, ^17^, ^18^, ^19^ kidney cancer,^10^, ^12^, ^19^, ^20^, ^21^ and lymphomas/leukemias.^10^, ^12^, ^21^, ^22^ The present study aimed to provide updated estimates of the risk of SPC after TC, as well as the risk of TC as SPC. In addition, we comprehensively explored the risks of TC as a first or SPC by sex, age, and time since first diagnosis. Evidence on the combination of TC with other cancers, as a first and second primary one, may help clarify whether the association is due to shared genetic or lifestyle risk factors, close anatomic proximity, treatment of the first cancer, or intensity of diagnostic activities.

## 2 MATERIALS AND METHODS

### 2.1 Study population

We analyzed data collected in 28 population‐based Italian cancer registries (CRs), covering over 22 million inhabitants (39% of the Italian population) (Appendix 1). All included CRs had been active for at least 10 years in the period of interest, that is 1998–2012.^3^

First cancer and SPC were classified using international classifications for topography and morphology (ICD10 and ICD‐O‐3).^23^, ^24^ We analyzed 36 cancer sites or types (Appendix 2), but we excluded non‐melanoma skin cancer, cases detected at autopsy, and those known from death certificate only or with follow‐up time equal to zero. Third or subsequent malignant tumors were very rare (0.3% of all subjects) and cases diagnosed at the age of 85 years or more were, therefore, excluded.^24^

Two cohorts were analyzed:

*Cohort 1 (SPC after TC)*: included patients with a TC diagnosis evaluated for the incidence of a SPC other than TC, with overall 276,100 person‐years of observation (216,431 in women and 59,669 in men).

*Cohort 2 (TC as SPC)*: included patients with cancers other than TC evaluated for TC incidence. TC cases diagnosed as a first tumor were excluded due to the international rules for multiple primaries definition adopted by Italian CRs.^24^, ^25^ Cohort 2 included 6,984,420 person‐years of observation (3,643,622 in women and 3,340,798 in men).

Observation started on the date of first cancer diagnosis and ended on the first date among: SPC, last known date of vital status, death, 31 December 2012, or the end of the most recent available year of full registration (Appendix 1).

### 2.2 Statistical analysis

Person‐years at risk (PY) were computed by first cancer site, histological type of TC, (i.e., papillary, follicular, medullary, and poorly differentiated including anaplastic), geographic area (North, Center, and South and Islands), sex, age group (0–4 years, …, 80–84 years), and calendar‐year group (1998–2002, 2003–2007, and 2008–2012). Observed cases included incident cancers reported to CRs during the above‐defined person‐years at risk. The expected number of cancer cases was computed by multiplying the cumulative person‐years of observation by the specific incidence rates for the strata in which person‐years were distributed. Observed SPC incidence among cancer patients was compared with expected numbers by means of standardized incidence ratio (SIR). Byar's approximation was applied to the exact Poisson distribution to calculate 95% confidence interval (CI).

SIRs were stratified by sex, follow‐up time (<2, 2–11, 12–35, 36–59, and 60+ months), and age at first cancer diagnosis (0–34, 35–54, and 55–84 years). This age stratification was chosen since the peak of TC incidence in Italy occurred in middle age, with approximately the same number of TC cases at ages 35–54 (17,043) and 55–84 (15,099) years (Appendix 1).

To minimize the impact of “intensive screening” in concurrence with the first cancer diagnosis,^24^, ^25^, ^26^ which may detect other pre‐existing tumors, observed and expected cases during the synchronous period (2 months) were shown only in the analysis by follow‐up duration. All the other SIRs were, therefore, computed excluding observed cases and person‐years in the first 2 months after the first tumor. Excess absolute risk (EAR) was computed (with 95% CIs) subtracting the expected number of subsequent cancers in the general population from observed number; the difference was then divided by the PY and the number of cancer cases in excess (or deficit) was expressed per 1,000 PY. All analyses were conducted using the “MP‐SIR” session of SEER*Stat 8.3.6.^27^

## 3 RESULTS

**FIGURE 1:**
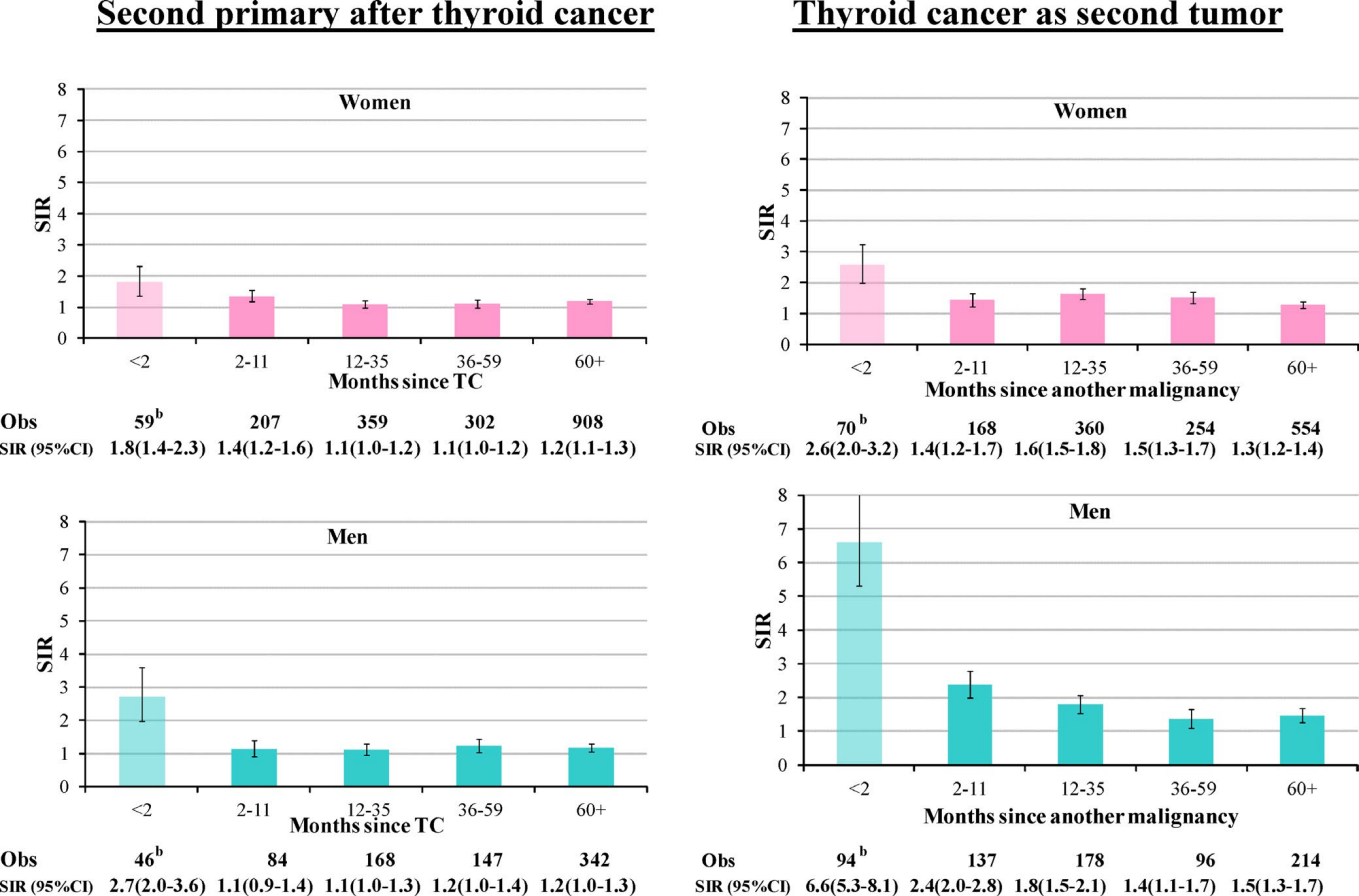
Risk^a^ of second primary cancer after thyroid cancer and risk of thyroid cancer as second tumor by time since the first tumor. Italy, 1998–2012. Obs, observed cases. ^a^Measured as standardized incidence ratio (SIR) and 95% confidence intervals (CIs). ^b^Excluded from subsequent analyses (4% of cancer subsequent to TC and 8% of TC after another malignancy)

The study populations included 1,406,694 patients who had been diagnosed with cancer below the age of 85 years, between 1998 and 2012 (38,535 patients with TC and 1,368,159 with any other primary cancer) (Appendix 1), followed for a maximum of 15 years (median follow‐up <7 years). After TC diagnosis, the SIR of other cancers diagnosed in <2 months‐period (i.e., synchronous) was high (1.8, 95% CI: 1.4–2.3 in women and 2.7, 95% CI: 2.0–3.6 in men), even if they represented only 4% of all the tumors (Figure 1). At 12–59 months after TC diagnosis, the SIRs in both sexes were between 1.1 and 1.3, and they remained 1.2 even 5 years after TC. The SIR for TC <2 months after other neoplasm (8% of all such cases) was 2.6 in women and 6.6 in men, and it gradually decreased up to 1.3 (95% CI: 1.2–1.4) in women and 1.5 (95% CI: 1.3–1.7) in men after 5 or more years since first cancer diagnosis (Figure 1).

In Italy, the overall SIR of second metachronous (i.e., diagnosed after >2 months since TC) cancers after TC was 1.16 (95% CI: 1.12–1.21) (Table 1). Notably, for patients with follicular or medullary TC the SIRs were not higher than the general population (SIRs = 1.06 and 0.95, respectively). For all cancers and most individual cancer types, SIRs after TC were consistent in men and women. Breast cancer represented 35% of all cancers diagnosed after TC in women (628/1776, SIR = 1.2, 1.1–1.3), while prostate was 24% of all cancers in men (SIR = 1.4, 1.2–1.6). After TC, no significant SIR increase emerged for cancers of colon–rectum (286 cases, SIR = 1.0), lung (204 cases, SIR 1.0), head and neck (41 cases SIR = 0.8), and female genital organs (endometrial SIR = 1.1, cervix 0.7 or ovary 1.2). Conversely, elevated SIRs were observed for cancers of the kidney (SIR = 2.2), soft tissue cancers (SIR = 2.1), and the combination of hemolymphopoietic neoplasms (SIR = 1.4), as well as for most of the hemolymphopoietic subtypes (SIR = 2.7 for acute lymphoid leukemia, 1.6 for follicular non‐Hodgkin lymphomas, 1.5 for chronic lymphoid leukemia, and 1.4 for myelomas).

**TABLE 1:** Risk^a^ of second primary cancer after thyroid cancer by cancer type and sex. Italy, 1998–2012

	Women (PY 216,431)				Men (PY 59,669)				Women and men (PY 276,100)			
	Obs	SIR	95%	CI	Obs	SIR	95%	CI	Obs	SIR	95%	CI
All but skin and thyroid, after TC	**1776**	**1.16**	**1.11**	**1.22**	**741**	**1.16**	**1.08**	**1.25**	**2517**	**1.16**	**1.12**	**1.21**
After papillary TC	1403	**1.19**	**1.13**	**1.26**	533	**1.19**	**1.10**	**1.30**	1936	**1.19**	**1.14**	**1.25**
After follicular TC	195	1.03	0.89	1.18	112	1.14	0.94	1.37	307	1.06	0.95	1.19
After medullary TC	57	1.02	0.77	1.32	40	0.87	0.62	1.19	97	0.95	0.77	1.16
After poorly differentiated^b^ TC	24	**2.07**	**1.32**	**3.08**	11	1.25	0.62	2.23	35	**1.71**	**1.19**	**2.38**
Second primary cancer (after TC)												
Head and neck	11	**0.5**	**0.3**	**0.9**	30	1.0	0.7	1.5	41	0.8	0.6	1.1
Oral cavity	5	0.5	0.1	1.1	3	0.4	0.1	1.2	8	**0.4**	**0.2**	**0.9**
Pharynx	1	**0.2**	**0.0**	**1.0**	4	0.5	0.1	1.4	5	**0.4**	**0.1**	**0.9**
Larynx	5	1.0	0.3	2.4	23	1.6	1.0	2.4	28	1.4	1.0	2.1
Esophagus	5	0.9	0.3	2.1	6	0.9	0.3	2.0	11	0.9	0.5	1.7
Stomach	59	1.0	0.8	1.3	36	1.2	0.8	1.6	95	1.1	0.9	1.3
Colon and rectum	204	1.0	0.9	1.2	82	1.0	0.8	1.2	286	1.0	0.9	1.1
Colon	140	1.0	0.8	1.2	64	1.1	0.9	1.4	204	1.0	0.9	1.2
Rectum	64	1.1	0.9	1.4	18	0.7	0.4	1.0	82	1.0	0.8	1.2
Liver	27	0.9	0.6	1.2	21	0.8	0.5	1.2	48	0.8	0.6	1.1
Gallbladder	16	0.7	0.4	1.2	4	0.7	0.2	1.7	20	0.7	0.4	1.1
Pancreas	59	1.2	0.9	1.6	11	0.6	0.3	1.1	70	1.1	0.8	1.3
Lung	116	**1.2**	**1.0**	**1.5**	88	0.9	0.7	1.1	204	1.0	0.9	1.2
Melanoma	45	1.0	0.8	1.4	19	1.3	0.8	2.0	64	1.1	0.8	1.4
Mesothelioma	4	1.0	0.3	2.7	8	2.1	0.9	4.2	12	1.6	0.8	2.7
Kaposi sarcoma	1	0.6	0.0	3.6	0	0.0	0.0	2.2	1	0.3	0.0	1.7
Bone and soft tissue	17	**1.8**	**1.1**	**2.9**	9	**2.5**	**1.1**	**4.7**	26	**2.0**	**1.3**	**3.0**
Soft tissue	13	1.8	1.0	3.1	8	**2.8**	**1.2**	**5.6**	21	**2.1**	**1.3**	**3.2**
Bone	4	2.0	0.5	5.0	1	1.2	0.0	6.9	5	1.8	0.6	4.1
Breast	628	**1.2**	**1.1**	**1.3**	1	0.7	0.0	3.8	629	**1.2**	**1.1**	**1.3**
Corpus uteri	93	1.1	0.9	1.4	0				93	1.1	0.9	1.4
Cervix uteri	18	0.7	0.4	1.1	0				18	0.7	0.4	1.1
Ovary	62	1.2	0.9	1.5	0				62	1.2	0.9	1.5
Prostate	0				178	**1.4**	**1.2**	**1.6**	178	**1.4**	**1.2**	**1.6**
Testis	0				6	1.6	0.6	3.5	6	1.6	0.6	3.5
Kidney and renal pelvis	69	**1.9**	**1.5**	**2.4**	59	**2.5**	**1.9**	**3.3**	128	**2.2**	**1.8**	**2.6**
Urinary bladder	53	1.2	0.9	1.6	64	1.0	0.7	1.2	117	1.1	0.9	1.3
Central nervous system	17	0.7	0.4	1.2	15	1.6	0.9	2.6	32	1.0	0.7	1.4
Hemolymphopoietic	171	**1.5**	**1.2**	**1.7**	62	**1.3**	**1.0**	**1.7**	233	**1.4**	**1.2**	**1.6**
Hodgkin lymphoma	8	1.2	0.5	2.3	3	1.1	0.2	3.3	11	1.2	0.6	2.1
Non‐Hodgkin lymphoma	72	1.3	1.0	1.6	26	1.2	0.8	1.8	98	**1.3**	**1.0**	**1.5**
Myeloma	36	**1.6**	**1.1**	**1.2**	9	1.1	0.5	2.0	45	**1.4**	**1.1**	**1.9**
CLL‐SLL	19	1.4	0.8	2.2	12	1.8	0.9	3.1	31	**1.5**	**1.0**	**2.2**
NHL, DLBC	16	0.9	0.5	1.5	6	1.0	0.4	2.1	22	0.9	0.6	1.4
NHL, Follicular	17	1.6	1.0	2.6	5	1.7	0.5	3.9	22	**1.6**	**1.0**	**2.5**
Acute L. Leukemia	7	**3.3**	**1.3**	**6.8**	1	1.3	0.0	7.0	8	**2.7**	**1.2**	**5.4**
Other and ill defined	101	1.1	0.9	1.3	42	1.2	0.9	1.6	143	1.1	0.9	1.3

PY, Person‐years; Obs, observed cases. NHL, Non‐Hodgkin lymphoma; CLL‐SLL, chronic lymphoid leukemia‐small lymphocytic lymphoma; DLBC, diffuse large B‐cell.

Statistical significant associations are highlighted in **bold**.

^a^ Measured as standardized incidence ratio (SIR) and 95% confidence intervals (CIs). Age 0–84 years; second primary cancers diagnosed <2 months after first one were excluded.

^b^ Poorly differentiated including anaplastic.

For younger TC patients (age < 35 years), SIRs of all subsequent cancer types were 1.53 (1.27–1.83) (Table 2), 1.45 in women and 1.93 in men (Appendix 3). SIR decreased with aging to 1.12 (1.06–1.17) in TC patients aged 55 years or more (Table 2). This age‐related pattern was present for hemolymphopoietic neoplasms (SIR = 2.0 below 35 years, 1.3 at ≥ 55 years) and for prostate (1.7 at age 35–54 and 1.4 at ≥ 55 years) (Appendix 3). Variation according to age was not observed in women for secondary breast cancer (SIR ~1.2 at all age groups), melanoma, colorectal, or kidney cancer (SIR ~2 in all age groups).

**TABLE 2:** Risk^a^ of second primary cancer after thyroid cancer (TC) by cancer type and age. Italy, 1998–2012

	Age at first cancer (TC)											
	0–34 years				35–54 years				55–84 years			
	Obs	SIR	95%	CI	Obs	SIR	95%	CI	Obs	SIR	95%	CI
All but skin and thyroid, after TC	**123**	**1.53**	**1.27**	**1.83**	**833**	**1.21**	**1.13**	**1.29**	**1561**	**1.12**	**1.06**	**1.17**
After papillary TC	102	**1.56**	**1.27**	**1.89**	683	**1.23**	**1.14**	**1.32**	1151	**1.15**	**1.08**	**1.22**
After follicular TC	13	1.51	0.80	2.58	80	1.11	0.88	1.38	214	1.03	0.90	1.18
After medullary TC	4	2.01	0.54	5.16	18	0.88	0.52	1.39	75	0.95	0.74	1.19
After poorly differentiated^b^ TC	1	9.19	0.12	51.14	2	1.00	0.11	3.61	32	**1.75**	**1.19**	**2.46**
Second primary cancer (after TC)^c^												
Head and neck	3	2.1	0.4	6.0	10	**0.5**	**0.3**	**1.0**	28	0.9	0.6	1.3
Stomach	1	0.6	0.0	3.5	19	1.0	0.6	1.6	75	1.1	0.9	1.4
Colon and rectum	9	1.9	0.9	3.6	58	0.8	0.6	1.0	219	1.1	0.9	1.2
Liver	1	1.5	0.0	8.6	5	0.4	0.1	1.0	42	0.9	0.7	1.2
Pancreas	0	0.0	0.0	4.5	19	1.3	0.8	2.1	51	1.0	0.7	1.3
Lung	3	1.2	0.2	3.5	58	1.2	0.9	1.5	143	1.0	0.8	1.2
Melanoma	14	**1.8**	**1.0**	**3.1**	18	0.7	0.4	1.1	32	1.3	0.9	1.8
Breast	36	1.2	0.9	1.7	295	**1.3**	**1.1**	**1.4**	298	**1.2**	**1.1**	**1.3**
Corpus uteri	5	2.5	0.8	5.8	45	1.4	1.0	1.8	43	0.9	0.6	1.2
Ovary	5	1.9	0.6	4.4	26	1.2	0.8	1.8	31	1.1	0.7	1.6
Prostate	0	0.0	0.0	6.8	38	**1.7**	**1.2**	**2.4**	140	**1.4**	**1.1**	**1.6**
Kidney and renal pelvis	4	2.2	0.6	5.7	47	**2.6**	**1.9**	**3.5**	77	**1.9**	**1.5**	**2.4**
Urinary bladder	1	0.6	0.0	3.2	39	**1.6**	**1.1**	**2.1**	77	0.9	0.7	1.2
Central nervous system	3	1.5	0.3	4.3	12	1.1	0.6	1.9	17	0.9	0.5	1.4
Hemolymphopoietic	19	**2.0**	**1.2**	**3.2**	71	**1.4**	**1.1**	**1.8**	143	**1.3**	**1.1**	**1.6**
Non‐Hodgkin lymphoma	6	1.5	0.6	3.3	25	1.0	0.6	1.5	67	**1.4**	**1.1**	**1.7**
Myeloma	1	2.0	0.0	11.1	13	1.7	0.9	2.8	31	1.4	0.9	1.9
Leukemia	7	**3.3**	**1.3**	**6.8**	28	**2.2**	**1.5**	**3.2**	44	**1.4**	**1.0**	**1.9**
												

Obs, observed cases.

Statistical significant associations are highlighted in **bold**.

^a^ Measured as standardized incidence ratio (SIR) and 95% confidence intervals (CIs). Men and women; second primary cancers diagnoses <2 months after first one were excluded.

^b^ Poorly differentiated including anaplastic.

^c^ Cancer types with >30 cases in men and women.

TC as SPC was diagnosed more frequently than in the general population: overall SIR = 1.49 (95% CI: 1.42–1.55), 1.42 in women and 1.67 in men (Table 3). SIR ranged between 1.35 for follicular TC and 1.61 for medullary TC. SIR of TC after female breast cancer was 1.3 (1.2–1.4) and increased SIRs were also found after acute lymphoid leukemia (SIR = 6.1), bone cancers (4.3), kidney cancers (3.2), Hodgkin lymphomas (2.8), head and neck cancers (2.1), melanoma (2.0), lung cancers (1.8), all hemolymphopoietic neoplasms (1.8), prostate (1.5), colorectal (1.4), and endometrial cancers (1.4) (Table 3).

**TABLE 3:** Risk^a^ of thyroid cancers (TC) as second tumor by first cancer type and sex. Italy, 1998–2012

	Women (PY 3,643,622)				Men (PY 3,340,798)				Women and Men (PY 6,984,420)			
	Obs	SIR	95%	CI	Obs	SIR	95%	CI	Obs	SIR	95%	CI
TC after all neoplasms, but skin and TC	1336	**1.42**	**1.34**	**1.49**	625	**1.67**	**1.54**	**1.80**	1961	**1.49**	**1.42**	**1.55**
Papillary TC, as second	1043	**1.44**	**1.36**	**1.53**	439	**1.80**	**1.64**	**1.98**	1482	**1.53**	**1.46**	**1.61**
Follicular TC, as second	117	**1.40**	**1.16**	**1.68**	56	1.26	0.95	1.64	173	**1.35**	**1.16**	**1.57**
Medullary TC, as second	58	**1.58**	**1.20**	**2.04**	42	**1.66**	**1.19**	**2.24**	100	**1.61**	**1.31**	**1.96**
Poorly differentiated^b^ TC, as second	41	1.37	0.99	1.86	33	**1.49**	**1.02**	**2.09**	74	**1.42**	**1.12**	**1.79**
Cancer types (first)												
Head and neck	20	**1.7**	**1.0**	**2.6**	53	**2.3**	**1.8**	**3.1**	73	**2.1**	**1.7**	**2.7**
Oral cavity	6	1.1	0.4	2.4	18	**4.0**	**2.4**	**6.3**	24	**2.4**	**1.5**	**3.6**
Pharynx	7	2.4	1.0	4.9	7	1.8	0.7	3.7	14	**2.1**	**1.1**	**3.4**
Larynx	7	2.1	0.8	4.3	28	**2.0**	**1.3**	**2.8**	35	**2.0**	**1.4**	**2.8**
Esophagus	2	2.1	0.2	7.6	1	0.8	0.0	4.2	3	1.3	0.3	3.9
Stomach	18	0.9	0.5	1.4	12	0.9	0.5	1.6	30	0.9	0.6	1.3
Colon and rectum	135	**1.3**	**1.1**	**1.5**	95	**1.7**	**1.4**	**2.1**	230	**1.4**	**1.3**	**1.6**
Colon	86	1.2	1.0	1.5	66	**1.7**	**1.4**	**2.2**	152	**1.4**	**1.2**	**1.6**
Rectum	49	**1.6**	**1.2**	**2.1**	29	**1.6**	**1.0**	**2.2**	78	**1.6**	**1.2**	**2.0**
Liver	8	1.5	0.7	3.0	5	0.8	0.3	1.8	13	1.1	0.6	1.9
Gallbladder	6	1.7	0.6	3.7	3	2.3	0.5	6.7	9	1.9	0.9	3.5
Pancreas	4	0.9	0.2	2.3	2	1.0	0.1	3.6	6	0.9	0.3	2.0
Lung	29	**1.8**	**1.2**	**2.5**	37	**1.8**	**1.2**	**2.4**	66	**1.8**	**1.4**	**2.2**
Melanoma	60	**1.5**	**1.2**	**2.0**	47	**3.6**	**2.6**	**4.8**	107	**2.0**	**1.7**	**2.5**
Mesothelioma	1	1.6	0.0	8.7	1	1.5	0.0	8.2	2	1.5	0.2	5.5
Kaposi sarcoma	2	1.9	0.2	7.0	3	1.9	0.4	5.4	5	1.9	0.6	4.4
Bone and soft tissue	21	**3.0**	**1.9**	**4.7**	7	2.3	0.9	4.7	28	**2.8**	**1.9**	**4.1**
Soft tissue	11	**2.2**	**1.1**	**3.9**	6	2.6	0.9	5.7	17	**2.3**	**1.3**	**3.7**
Bone	10	**5.6**	**2.7**	**10.3**	1	1.3	0.0	7.5	11	**4.3**	**2.2**	**7.8**
Breast	583	**1.3**	**1.2**	**1.4**	1	0.8	0.0	4.4	584	**1.3**	**1.2**	**1.4**
Corpus uteri	87	**1.4**	**1.1**	**1.7**	0				87	**1.4**	**1.1**	**1.7**
Cervix uteri	32	1.2	0.8	1.7	0				32	1.2	0.8	1.7
Ovary	33	1.2	0.8	1.7	0				33	1.2	0.8	1.7
Prostate	0				147	**1.5**	**1.3**	**1.8**	147	**1.5**	**1.3**	**1.8**
Testis	0				12	1.8	0.9	3.2	12	1.8	0.9	3.2
Kidney and renal pelvis	67	**3.2**	**2.5**	**4.0**	54	**3.1**	**2.4**	**4.1**	121	**3.2**	**2.6**	**3.8**
Urinary bladder	32	1.2	0.8	1.6	62	1.1	0.9	1.4	94	1.1	0.9	1.4
Central nervous system	15	**2.4**	**1.3**	**3.9**	6	2.3	0.8	5.0	21	**2.3**	**1.5**	**3.6**
Hemolymphopoietic	128	**1.8**	**1.5**	**2.1**	55	**1.7**	**1.2**	**2.2**	183	**1.8**	**1.5**	**2.0**
Hodgkin lymphoma	25	**2.6**	**1.7**	**3.8**	13	**3.5**	**1.9**	**6.0**	38	**2.8**	**2.0**	**3.9**
Non‐Hodgkin lymphoma	69	**1.9**	**1.5**	**2.4**	22	1.4	0.9	2.1	91	**1.7**	**1.4**	**2.1**
Myeloma	10	1.0	0.5	1.9	5	1.1	0.4	2.6	15	1.0	0.6	1.7
CLL‐SLL	9	1.0	0.4	1.9	6	1.0	0.4	2.3	15	1.0	0.6	1.7
NHL, DLBC	17	**2.0**	**1.2**	**3.2**	5	1.3	0.4	3.1	22	**1.8**	**1.1**	**2.7**
NHL, Follicular	19	**2.4**	**1.5**	**3.8**	3	1.1	0.2	3.2	22	**2.1**	**1.3**	**3.2**
Acute L. Leukemia	4	3.4	0.9	8.7	6	**13.2**	**4.8**	**28.7**	10	**6.1**	**2.9**	**11**.**2**
Other and ill defined	53	1.3	1.0	1.7	22	1.2	0.7	1.8	75	1.3	1.0	1.6

PY, Person‐years; Obs, observed cases; NHL, Non‐Hodgkin lymphoma; CLL‐SLL, chronic lymphoid leukemia‐small lymphocytic lymphoma; DLBC, diffuse large B‐cell.

Statistical significant associations are highlighted in **bold**.

^a^ Measured as standardized incidence ratio (SIR) and 95% confidence intervals (CIs). Age 0–84 years; second primary cancers diagnosed <2 months after first one were excluded.

^b^ Poorly differentiated including anaplastic.

The most elevated SIRs for TC as SPC were observed when primary cancer was diagnosed below age 35 years (SIR = 2.69, 2.25–3.19) (Table 4), more elevated in men (3.6, 2.6–5.0) than in women (2.4, 2.0–3.0) (Appendix 4). In this age group, first tumors were hemolymphopoietic cancers in 52 out of 133 (39%) patients with SIR for TC = 4.3 overall and 3‐fold higher than expected for all major hemolymphopoietic cancers. The 35–54 and 55–84 years age groups showed similar SIRs for TC as SPC (1.50 and 1.41, respectively), after breast (1.2 and 1.4, respectively), and corpus uteri cancers (1.3 and 1.4). SIRs for TC decreased with age after colorectal cancer and kidney cancer (Table 4).

**TABLE 4:** Risk^a^ of thyroid cancers (TC) as second tumor by first cancer type and age. Italy, 1998–2012

	Age at first cancer											
	0–34 years				35–54 years				55–84 years			
	Obs	SIR	95%	CI	Obs	SIR	95%	CI	Obs	SIR	95%	CI
TC after all neoplasms, but skin and TC	133	**2.69**	**2.25**	**3.19**	653	**1.50**	**1.38**	**1.61**	1175	**1.41**	**1.33**	**1.49**
Papillary TC, as second	114	**2.67**	**2.21**	**3.21**	550	**1.51**	**1.38**	**1.64**	818	**1.46**	**1.37**	**1.57**
Follicular TC, as second	8	**2.52**	**1.08**	**4.96**	42	1.32	0.95	1.79	123	**1.32**	**1.10**	**1.57**
Medullary TC, as second	2	2.03	0.23	7.34	24	**1.68**	**1.08**	**2.51**	74	**1.58**	**1.24**	**1.98**
Poorly differentiated^b^ TC, as second	1	10.09	0.13	56.14	8	1.95	0.84	3.85	65	**1.36**	**1.05**	**1.73**
Cancer types (first)^c^												
Head and neck	1	1.6	0.0	9.0	23	**2.2**	**1.4**	**3.3**	49	**2.1**	**1.5**	**2.8**
Stomach	0	0.0	0.0	7.9	4	0.5	0.1	1.3	26	1.1	0.7	1.5
Colon and rectum	3	2.0	0.4	5.9	57	**1.7**	**1.3**	**2.2**	170	**1.4**	**1.2**	**1.6**
Lung	1	2.6	0.0	14.4	20	**2.7**	**1.7**	**4.2**	45	**1.5**	**1.1**	**2.0**
Melanoma	13	1.9	1.0	3.2	52	**2.2**	**1.6**	**2.9**	42	**1.9**	**1.4**	**2.6**
Breast	13	1.4	0.8	2.5	256	**1.2**	**1.1**	**1.4**	315	**1.4**	**1.2**	**1.5**
Corpus uteri	2	0.7	0.1	2.7	27	1.3	0.9	1.9	60	**1.4**	**1.1**	**1.8**
Cervix uteri	0	0.0	0.0	6.6	19	1.2	0.7	1.9	11	1.3	0.7	2.4
Ovary	2	0.9	0.1	3.3	18	1.4	0.9	2.3	13	1.0	0.5	1.7
Prostate	0	0.0	0.0	666.9	7	1.8	0.7	3.8	140	**1.5**	**1.3**	**1.8**
Kidney and renal pelvis	7	**7.2**	**2.9**	**14.8**	47	**4.2**	**3.1**	**5.6**	67	**2.6**	**2.0**	**3.2**
Urinary bladder	3	2.6	0.5	7.5	16	1.0	0.6	1.7	75	1.1	0.9	1.4
Hemolymphopoietic	52	**4.3**	**3.2**	**5.6**	60	**1.8**	**1.4**	**2.3**	71	1.2	0.9	1.5
Hodgkin lymphoma	28	**4.5**	**3.0**	**6.6**	8	1.6	0.7	3.2	2	0.9	0.1	3.1
Non‐Hodgkin lymphoma	12	**3.1**	**1.6**	**5.5**	33	**1.9**	**1.3**	**2.6**	46	**1.5**	**1.1**	**2.0**
Leukemia	12	**5.8**	**3.0**	**10.2**	15	**2.1**	**1.2**	**3.5**	12	0.8	0.4	1.4

Obs, observed cases.

Statistical significant associations are highlighted in **bold**.

^a^ Measured as standardized incidence ratio (SIR) and 95% confidence intervals (CIs). Men and women; second primary cancers diagnosed <2 months after first one were excluded.

^b^ Poorly differentiated including anaplastic.

^c^ Cancer types with ≥30 cases in men and women.

EAR after TC for all cancers was 1.28 per 1,000 PY (Table 5), 0.43 per 1,000 PY for breast cancer, 0.25 per 1,000 PY for kidney cancer, and 0.19 per 1,000 PY for prostate cancer. All other cancers showed EAR < 0.1 per 1,000 PY. EAR of TC as a SPC was 0.09 per 1,000 PY overall, 0.51 per 1,000 PY after bone, 0.36 after kidney, 0.35 after acute lymphoid leukemia, 0.31 after Hodgkin lymphoma, 0.23 after oral cavity and brain, 0.22 after melanoma and follicular NHL (Table 5).

**TABLE 5:** Excess absolute risk (EAR) of second primary cancer in thyroid cancer (TC) patients and TC as second tumor by first cancer type^a^. Italy, 1998–2012

Cancer types	Second primary cancer after TC			TC as second tumor		
	EAR per 1000 py	95%	CI	EAR per 1000 py	95%	CI	
All other neoplasms, but skin and thyroid	**1.28**	**0.79**	**1.77**	**0.09**	**0.08**	**0.11**	
Papillary TC	**1.35**	**0.84**	**1.85**	**0.07**	**0.06**	**0.09**	
Follicular TC	0.58	−0.92	2.09	**0.01**	**0.00**	**0.01**	
Medullary TC	−0.47	−3.16	2.22	**0.01**	**0.00**	**0.01**	
Poorly differentiated^b^ TC	**9.37**	**0.07**	**18.67**	0.00	0.00	0.01	
Head and neck	−0.01	−0.03	0.01	**0.16**	**0.08**	**0.25**	
Oral cavity	**−0.04**	**−0.07**	**0.00**	**0.23**	**0.04**	**0.43**	
Pharynx	−0.03	−0.06	0.00	0.17	−0.04	0.39	
Larynx	0.03	−0.02	0.08	**0.13**	**0.02**	**0.24**	
Esophagus	0.00	−0.04	0.03	0.05	−0.25	0.35	
Stomach	0.03	−0.07	0.12	−0.01	−0.09	0.06	
Colon and rectum	0.01	−0.08	0.09	**0.07**	**0.03**	**0.12**	
Colon	0.02	−0.12	0.17	**0.06**	**0.02**	**0.11**	
Rectum	−0.01	−0.10	0.08	**0.10**	**0.02**	**0.17**	
Liver	−0.04	−0.11	0.04	0.02	−0.11	0.14	
Gallbladder	−0.03	−0.08	0.02	0.15	−0.11	0.41	
Pancreas	0.01	−0.07	0.10	−0.01	−0.21	0.19	
Lung	0.03	−0.11	0.17	**0.12**	**0.04**	**0.20**	
Melanoma	0.02	−0.06	0.10	**0.22**	**0.12**	**0.31**	
Mesothelioma	0.02	−0.02	0.05	0.08	−0.36	0.53	
Kaposi sarcoma	−0.01	−0.02	0.01	0.11	−0.15	0.37	
Bone and soft tissue	**0.02**	**0.00**	**0.05**	**0.30**	**0.10**	**0.50**	
Soft tissue	**0.04**	**0.00**	**0.08**	0.22	0.00	0.45	
Bone	0.01	−0.01	0.03	**0.51**	**0.08**	**0.95**	
Breast	**0.43**	**0.19**	**0.67**	**0.09**	**0.05**	**0.12**	
Corpus uteri	0.03	−0.06	0.13	0.10	0.00	0.20	
Cervix uteri	−0.03	−0.08	0.02	0.06	−0.11	0.22	
Ovary	0.03	−0.04	0.11	0.05	−0.10	0.21	
Prostate	**0.19**	**0.07**	**0.31**	**0.06**	**0.02**	**0.09**	
Testis	0.01	−0.01	0.03	0.08	−0.04	0.19	
Kidney and renal pelvis	**0.25**	**0.15**	**0.35**	**0.36**	**0.25**	**0.47**	
Urinary bladder	0.03	−0.08	0.13	0.02	−0.02	0.06	
Central nervous system	0.00	−0.06	0.05	**0.23**	**0.03**	**0.43**	
Hemolymphopoietic	**0.04**	**0.02**	**0.07**	**0.13**	**0.08**	**0.19**	
Hodgkin lymphoma	0.01	−0.03	0.04	**0.31**	**0.13**	**0.48**	
Non‐Hodgkin lymphoma	0.07	−0.02	0.17	**0.14**	**0.06**	**0.22**	
Myeloma	0.05	−0.01	0.11	0.01	−0.13	0.14	
CLL‐SLL	0.04	−0.01	0.08	0.00	−0.12	0.12	
NHL, DLBC	0.00	−0.05	0.04	0.14	−0.03	0.32	
NHL, Follicular	0.03	−0.01	0.07	**0.22**	**0.01**	**0.44**	
Acute L. Leukemia	0.02	0.00	0.04	**0.35**	**0.07**	**0.63**	
Other and ill defined	0.05	−0.07	0.16	0.04	−0.02	0.11	

CI, confidence intervals. NHL, Non‐Hodgkin lymphoma; CLL‐SLL, chronic lymphoid leukemia‐small lymphocytic lymphoma; DLBC, diffuse large B‐cell.

Statistical significant associations are highlighted in **bold**.

^a^ Men and women, age 0–84 years; second primary cancers diagnosed <2 months after first one were excluded.

^b^ Poorly differentiated including anaplastic.

## 4 DISCUSSION

In Italy, TC patients have a 16% increased risk of experiencing a SPC, in comparison with the general population, slightly more than the corresponding figure in the United States (10%)^14^, ^21^ and South Korea (6%),^28^ but less than in Denmark (31%),^10^ Japan (44%),^11^ and Switzerland (58% in men and 36% in women).^13^ SIR in TC patients diagnosed in 1998–2012 corresponded to an excess incidence rate above 1 per 1,000 patients per year (i.e., 100/100,000). In addition, SIR of TC diagnosis after another cancer was 1.49, consistent with estimates in the United States (+50%).^12^

Other authors have reported higher risks of papillary TC than of follicular TC after several neoplasms, in particular renal or breast cancers and leukemias/lymphomas.^19^ However, in the present study, risks of SPC after papillary TC and of papillary TC as SPC are only slightly higher than corresponding risks for all TC (i.e., all other TC). Most notably, although rare, our data suggested that follicular or medullary TC were not associated with an excess of all SPC, while, as SPC, these two TC subtypes had similar SIRs than papillary or other TC. On the other hand, numbers for such types are quite small and possible associations, for example, as those in multiple endocrine neoplasia for medullary TC, may have not been evidenced.

Several studies reported an elevated risk of SPCs from the use of radioactive iodine (RAI) therapy in TC patients,^29^, ^30^ in particular in young patients.^31^ This may explain the elevated incidence of acute lymphoid leukemia and other hemolymphopoietic neoplasms after TC treated with RAI.^30^ However, increased risks after TC have been reported for different lymphoid and hematological neoplasms,^32^, ^33^ as well as for solid tumors.^30^ In three large cohorts of TC cases diagnosed until 1995,^34^ an increased risk of several solid tumors and leukemia's was found with increasing cumulative activity of administered iodine‐131 (^131^I). However, when contrasting those exposed and not exposed to ^131^I, significantly increased risks of SPC were only seen for bone and soft tissue cancers.^34^ The association between soft tissue sarcomas and TC (as a first or second neoplasm) that emerged in our study is consistent with recent results reported in the USA.^12^, ^14^

Despite the methodological flaw comparing SIRs after TC and for TC as SPC, the bidirectional association between TC and hemolymphopoietic, kidney, and bone/soft tissue cancers is remarkable and consistent across sex and age groups. The highest SIRs were observed for bone cancer and acute lymphoid leukemia after TC, although with broad CI. Moreover, there were moderate bidirectional associations between thyroid and prostate (1.4 after TC and 1.5 of TC as SPC) or breast cancer (1.2 and 1.2). Notably in our study SIR of breast cancer after TC was 1.2, did not change with time since TC diagnosis, and was similar to what has been reported in Europe^10^ and USA,^21^ but lower than in Japan (2.0)^11^ and Korea (2.5).^35^

The consistent associations found between TC and other tumors may suggest the contribution of common risk factors, such as obesity, including genetic predisposition.^36^, ^37^ Nevertheless, in the present study the association of TC with the two most common overweight‐related neoplasms (i.e., colorectal and endometrial cancers) was limited, if any, and in line with previous results from comparable population‐based studies.^10^, ^12^, ^14^ For some cancer types (e.g., breast or kidney cancer), the link with TC consistently persisted beyond 5 years since diagnosis^14^, ^19^, ^27^ and can be driven, at least in part, by genetic predisposition and treatment exposures.^16^, ^36^

To disentangle potential biological associations between first and subsequent tumors seems beyond the capability of CR data, since they are probably mediated by complex interactions between genetic predispositions, lifestyle, and intensity of surveillance.

Even if a role of previous radiotherapy on thyroid gland (a radiosensitive organ) was a possible explanation of a small proportion of excess risk for TC as SPC, the finding of a 50% excess risk for TC as SPC may largely be explained by increased medical surveillance in oncologic patients and be accompanied by overdiagnosis. In fact, in a context of overdiagnosis estimated to be at least 70% of TC in Italy^3^ as elsewhere,^9^ most of the excess risk of TC as SPC (but possibly also of SPC after TC) may be due to the intensity of diagnostic activities in cancer patients.^7^, ^38^ In our study, the increased risk of TC after upper aerodigestive tract cancers (SIR = 2.1, Table 3), but not the opposite (SIR = 0.8, Table 1), supports the relationship between TC and diagnostic procedures of the neck. In Korea, the country with the highest TC incidence worldwide, SIRs for kidney cancer after TC increased with calendar period and paralleled TC increase in the general population.^28^ For kidney cancer, the impact of enhanced screening (i.e., through abdominal ultrasound and imaging) has also been reported both in Korea^39^ and elsewhere.^40^, ^41^

### 4.1 Strengths and weaknesses

The major strengths of this study are the population‐based design, the availability of the largest well‐documented TC series (>38,500 cases) in Europe, and the corresponding cohort of patients with other cancer types (1.4 million cases). Few other studies,^10^, ^11^, ^14^, ^32^ could similarly explore the bidirectional association between TC and other cancers. To the best of our knowledge, only another study has compared the association of TC with other cancers using population‐based data with specific attention to tumor histology and latency.^19^ Our study is the first one capable of computing SIR separately for different TC histological types (i.e., follicular, medullary, and poorly differentiated) and more than 30 cancer types as a first or SPC.

Among the limitations, it should be mentioned that although Italian CRs were considered complete and accurate and cover one third of the population,^42^ they do not cover the whole country. They may miss part of the substantial TC incidence variability among Italian regions.^3^ Moreover, international comparisons of SIRs of second cancers suffer from the lack of a standardized cut‐off between metachronous (e.g., subsequent) and synchronous tumors. In the present study, we excluded cancers occurring in the 2 months after diagnosis (4% of those diagnosed after TC and 8% of TC as SPC). Their inclusion would have had a negligible impact for the overall SIR estimates. The same definition was used by some authors,^21^, ^28^, ^43^ but other cut‐offs were also used (such as 6 months,^13^, ^26^ 12 months,^12^, ^17^ or 5 years^14^), suggesting the need of caution in comparisons between studies. Unfortunately, Italian cancer registries do not systematically collect information on stage at diagnosis or treatment (e.g., RAI), and we could not take these important factors into account.^12^, ^34^, ^44^ The length of follow‐up (<15 years, median <7 years) precluded the assessment of longer term incidence of SPC. Finally, as a result of the large number of comparisons made, some SIRs may be spuriously statistically significant, calling for caution in the interpretation of results.

## 5 PUBLIC HEALTH CONSEQUENCES AND CONCLUSIONS

With the growing number of people living after a cancer diagnosis, the number of those developing a SPC will also increase, generating further medical and financial burdens for patients, families, and society.^45^ Our findings may have potential implications for screening other neoplasms in patients with certain types of malignancies. In particular, TC patients need a comprehensive support, including surveillance for treatment side effects. However, overdiagnosis and overtreatment of TC should be avoided, particularly among younger women.^46^, ^47^

## CONFLICT OF INTEREST

The authors have declared no competing interests.

## ETHICS APPROVAL AND CONSENT TO PARTICIPATE

The Italian legislation identifies regional health authorities as collectors of personal data for surveillance purposes without explicit individual consent. The approval of a research ethic committee is not required, since this study is a descriptive analysis of anonymous aggregate data without any direct or indirect intervention on patients (Decreto del Presidente del Consiglio dei Ministri, 3/3/2017, Identificazione dei sistemi di sorveglianza e dei registri di mortalità, di tumori e di altre patologie, 17A03142, GU Serie Generale n.109 del 12‐05‐2017). Available at: https://www.gazzettaufficiale.it/eli/id/2017/05/12/17A03142/sg, last access: 16/08/2021.

## Supporting information

Appendix S1‐S4

## Data Availability

Research data are available from the corresponding author upon reasonable request, in agreement with AIRTUM guidelines.
