# Supplementary material for: Risk of thyroid as a first or second primary cancer. A population‐based study in Italy, 1998–2012
Source: Cancer Med. 2021 Sep 17;10(19):6855–67. doi: 10.1002/cam4.4193 (PMC8495271; doi:10.1002/cam4.4193)
Supplement: Supplementary file 1 — Appendix S1‐S4 [file CAM4-10-6855-s001.docx]

**Appendix 1. Populations included in the study, observation period, and number of all^a,b^ and thyroid cancers patients by cancer registry, sex, age, and period. Italy, 1998-2012**

| **Registry/area** | **Population**  **2008 per million** | **Period** | **N. of cases**  **(1998-2012)** | |
| --- | --- | --- | --- | --- |
|  |  |  | **All cancers** | **Thyroid** |
| **All registries** | **22.36** |  | **1,368,159** | **38,535** |
|  |  |  |  |  |
| Alto Adige | 0.48 | 1998-2010 | 27,071 | 352 |
| Biella | 0.18 | 1998-2010 | 17,946 | 318 |
| Brescia | 1.08 | 1999-2008 | 53,275 | 1,665 |
| Catania-Messina-Enna | 1.86 | 2003-2012 | 75,161 | 4,204 |
| Ferrara | 0.34 | 1998-2011 | 31,212 | 1,124 |
| Firenze Prato | 1.16 | 1998-2008 | 69,077 | 1,376 |
| Friuli Venezia Giulia | 1.17 | 1998-2010 | 88,814 | 1,510 |
| Genova | 0.83 | 1998-2009 | 64,578 | 1,275 |
| Latina | 0.51 | 1998-2011 | 26,733 | 1,616 |
| Mantova | 0.39 | 1999-2010 | 24,637 | 762 |
| Milano | 1.19 | 1999-2010 | 91,843 | 1,415 |
| Modena | 0.65 | 1998-2012 | 50,973 | 2,083 |
| Napoli | 1.15 | 1998-2012 | 38,938 | 1,270 |
| Nuoro | 0.21 | 2003-2012 | 9,023 | 578 |
| Palermo | 1.21 | 2003-2012 | 48,024 | 1,753 |
| Parma | 0.4 | 1998-2012 | 35,515 | 1,344 |
| Ragusa | 0.56 | 1998-2012 | 22,456 | 794 |
| Reggio Emilia | 0.49 | 1998-2012 | 36,712 | 1,358 |
| Romagna | 1.13 | 1998-2012 | 92,805 | 3,311 |
| Salerno | 1.07 | 1998-2009 | 44,596 | 1,492 |
| Sassari | 0.46 | 1998-2011 | 25,422 | 1,023 |
| Siracusa | 0.39 | 1999-2012 | 20,179 | 716 |
| Sondrio | 0.18 | 1998-2011 | 12,460 | 214 |
| Torino | 0.85 | 1998-2012 | 69,527 | 1,272 |
| Trento | 0.5 | 1998-2010 | 28,240 | 584 |
| Umbria | 0.84 | 1998-2011 | 64,077 | 1,397 |
| Varese | 0.83 | 1998-2012 | 60,909 | 1,080 |
| Veneto | 2.26 | 1998-2009 | 137,956 | 2,649 |
|  |  |  |  |  |
| **Sex** |  |  |  |  |
| Women |  |  | 630,102 | 29,424 |
| Men |  |  | 738,057 | 9,111 |
|  |  |  |  |  |
| **Age** |  |  |  |  |
| 0-34 years |  |  | 38,540 | 6,393 |
| 35-54 years |  |  | 211,727 | 17,043 |
| 55-84 years |  |  | 1,117,892 | 15,099 |
| **Period** |  |  |  |  |
| 1998-2002 |  |  | 435,584 | 8,657 |
| 2003-2007 |  |  | 548,198 | 15,636 |
| 2008-2012 |  |  | 384,377 | 14,242 |
|  |  |  |  |  |

^a^Age 0-84 years.

^b^Excluded non-melanoma skin cancers and thyroid cancers.

**Appendix 2. Classification of cancer types based on ICD10 and ICD-O-3 morphology**

|  |  |  |
| --- | --- | --- |
|  | **ICD10** | **ICDO3 morphology** |
| **All but skin and thyroid, after TC** | **C00-43, 47-72,74-80** |  |
|  |  |  |
| Thyroid cancer, Papillary | C73 | 8050, 8052, 8260, 8263, 8340-8344,  8350, 8450 |
| Thyroid cancer, Follicular | C73 | 8290, 8330-8335, |
| Thyroid cancer, Medullary | C73 | 8246,8345-8347,8510 |
| Thyroid cancer, Poorly differentiated**^a^** TC | C73 | 8012,8020-8035, 8190,8337 |
| **Cancer types** |  |  |
| Head and neck | C01-14,30-32 |  |
| *Oral cavity* | *C01-09* |  |
| *Pharynx* | *C10-13* |  |
| *Larynx* | *C32* |  |
| Oesophagus | C15 |  |
| Stomach | C16 |  |
| Colon and rectum | C18-21 |  |
| *Colon* | C18 |  |
| *Rectum* | C19-20 |  |
| Liver | C22 |  |
| Gallbladder | C23-24 |  |
| Pancreas | C25 |  |
| Lung | C33-34 |  |
| Skin Melanoma | C43 |  |
| Mesothelioma | C45 |  |
| Kaposi Sarcoma | C46 |  |
| Bone and soft tissue | C40-41,47,49 |  |
| *Bone* | *C40-41* |  |
| *Connective and soft tissue* | *C47,49* |  |
| Female Breast | C50 |  |
| Corpus uteri | C54 |  |
| Cervix uteri | C53 |  |
| Ovary | C56 |  |
| Prostate | C61 |  |
| Testis | C62 |  |
| Kidney and renal pelvis | C64-66,68 |  |
| Urinary bladder | C67,D090,D303,D414 |  |
| Central nervous system | C70-72 |  |
| Hemo-lymphopoietic | C81-96 |  |
| *Hodgkin lymphoma* |  | *M9650-96670* |
| *Non-Hodgkin lymphoma* | *C82-85,96* |  |
| *Myeloma* |  | *M9731-9734* |
| *CLL-SLL* |  | *M9670,9823* |
| *NHL, DLBC* |  | *M9678-9684* |
| *NHL, Follicular* |  | *M9675,9690-9698* |
| *Acute L.Leukemia* |  | *M9727-9729,9835-9837* |
|  |  |  |

^a^Poorly differentiated including anaplastic.

**Appendix 3 (Table 2 extended). Risk^a^ of second primary after thyroid cancer by cancer type, sex, and age. Italy, 1998-2012**

|  | **0-34 years** | | | | **35-54 years** | | | | **55-84 years** | | | |
| --- | --- | --- | --- | --- | --- | --- | --- | --- | --- | --- | --- | --- |
| **WOMEN** | **Obs** | **SIR** | **95 %** | **CI** | **Obs** | **SIR** | **95 %** | **CI** | **Obs** | **SIR** | **95 %** | **CI** |
| **All but skin and thyroid, after TC** | **96** | **1.45** | **1.17** | **1.77** | **638** | **1.17** | **1.08** | **1.27** | **1042** | **1.14** | **1.07** | **1.21** |
| After Papillary TC | 81 | **1.51** | **1.20** | **1.87** | 530 | **1.19** | **1.09** | **1.29** | 792 | **1.17** | **1.09** | **1.25** |
| After Follicular TC | 11 | 1.42 | 0.71 | 2.54 | 56 | 1.05 | 0.79 | 1.36 | 128 | 0.99 | 0.83 | 1.18 |
| After Medullary TC | 0 | 0.00 | 0.00 | 3.01 | 11 | 0.89 | 0.44 | 1.58 | 46 | 1.09 | 0.79 | 1.45 |
| After Poorly differentiated**^b^** TC | 1 | 9.78 | 0.13 | 54.44 | 1 | 0.63 | 0.01 | 3.49 | 22 | **2.22** | **1.39** | **3.36** |
|  |  |  |  |  |  |  |  |  |  |  |  |  |
| **Second primary (after TC)^c^** |  |  |  |  |  |  |  |  |  |  |  |  |
| Head and neck | 0 | 0.0 | 0.0 | 4.7 | 3 | 0.4 | 0.1 | 1.1 | 8 | 0.6 | 0.3 | 1.3 |
| Stomach | 1 | 0.9 | 0.0 | 4.9 | 11 | 0.9 | 0.4 | 1.6 | 47 | 1.1 | 0.8 | 1.4 |
| Colon and rectum | 8 | 2.0 | 0.9 | 4.0 | 44 | 0.8 | 0.6 | 1.1 | 153 | 1.1 | 0.9 | 1.3 |
| Liver | 1 | 3.5 | 0.0 | 19.4 | 1 | 0.2 | 0.0 | 1.0 | 25 | 1.0 | 0.6 | 1.4 |
| Pancreas | 0 | 0.0 | 0.0 | 6.4 | 15 | 1.5 | 0.8 | 2.4 | 44 | 1.2 | 0.8 | 1.6 |
| Lung | 2 | 1.2 | 0.1 | 4.3 | 35 | 1.2 | 0.9 | 1.7 | 79 | 1.2 | 1.0 | 1.5 |
| Melanoma | 11 | 1.8 | 0.9 | 3.2 | 13 | 0.7 | 0.3 | 1.1 | 21 | 1.2 | 0.7 | 1.9 |
| Breast | 36 | 1.2 | 0.9 | 1.7 | 295 | **1.3** | **1.1** | **1.4** | 297 | **1.2** | **1.1** | **1.3** |
| Corpus uteri | 5 | 2.5 | 0.8 | 5.8 | 45 | 1.4 | 1.0 | 1.8 | 43 | 0.9 | 0.6 | 1.2 |
| Ovary | 5 | 1.9 | 0.6 | 4.4 | 26 | 1.2 | 0.8 | 1.8 | 31 | 1.1 | 0.7 | 1.6 |
| Kidney and renal pelvis | 2 | 1.8 | 0.2 | 6.6 | 22 | **2.0** | **1.2** | **3.0** | 45 | **1.9** | **1.4** | **2.5** |
| Urinary bladder | 0 | 0.0 | 0.0 | 4.2 | 19 | **1.7** | **1.0** | **2.6** | 34 | 1.1 | 0.7 | 1.5 |
| Central nervous system | 1 | 0.7 | 0.0 | 3.9 | 6 | 0.8 | 0.3 | 1.7 | 10 | 0.7 | 0.3 | 1.3 |
| Hemo-lymphopoietic | 13 | **2.0** | **1.0** | **3.3** | 52 | **1.5** | **1.1** | **1.9** | 106 | **1.4** | **1.2** | **1.7** |
|  |  |  |  |  |  |  |  |  |  |  |  |  |
| **MEN** |  |  |  |  |  |  |  |  |  |  |  |  |
| **All but skin and thyroid, after TC** | 27 | **1.93** | **1.27** | **2.81** | 195 | **1.34** | **1.16** | **1.55** | 519 | 1.08 | 0.99 | 1.18 |
| After Papillary TC | 21 | **1.79** | **1.11** | **2.74** | 153 | **1.39** | **1.18** | **1.63** | 359 | 1.11 | 1.00 | 1.23 |
| After Follicular TC | 2 | 2.29 | 0.26 | 8.26 | 24 | 1.26 | 0.81 | 1.87 | 86 | 1.09 | 0.88 | 1.35 |
| After Medullary TC | 4 | **5.21** | **1.40** | **13.33** | 7 | 0.86 | 0.35 | 1.78 | 29 | 0.78 | 0.53 | 1.13 |
| After Poorly differentiated**^b^** TC | 0 | 0.00 | 0.00 | 555.76 | 1 | 2.43 | 0.03 | 13.55 | 10 | 1.19 | 0.57 | 2.19 |
|  |  |  |  |  |  |  |  |  |  |  |  |  |
| **Second primary (after TC)^c^** |  |  |  |  |  |  |  |  |  |  |  |  |
| Head and neck | 3 | 4.4 | 0.9 | 13.0 | 7 | 0.7 | 0.3 | 1.4 | 20 | 1.1 | 0.7 | 1.7 |
| Stomach | 0 | 0.0 | 0.0 | 8.5 | 8 | 1.3 | 0.6 | 2.6 | 28 | 1.2 | 0.8 | 1.7 |
| Colon and rectum | 2 | 1.7 | 0.2 | 6.2 | 14 | 0.7 | 0.4 | 1.2 | 66 | 1.0 | 0.8 | 1.3 |
| Liver | 0 | 0.0 | 0.0 | 10.2 | 4 | 0.7 | 0.2 | 1.7 | 17 | 0.8 | 0.5 | 1.3 |
| Pancreas | 0 | 0.0 | 0.0 | 14.7 | 4 | 1.0 | 0.3 | 2.5 | 7 | 0.5 | 0.2 | 1.0 |
| Lung | 1 | 1.2 | 0.0 | 6.6 | 23 | 1.1 | 0.7 | 1.7 | 64 | 0.8 | 0.6 | 1.0 |
| Melanoma | 3 | 2.2 | 0.5 | 6.5 | 5 | 0.9 | 0.3 | 2.1 | 11 | 1.4 | 0.7 | 2.5 |
| Prostate | 0 | 0.0 | 0.0 | 6.8 | 38 | **1.7** | **1.2** | **2.4** | 140 | **1.4** | **1.1** | **1.6** |
| Kidney and renal pelvis | 2 | 2.9 | 0.3 | 10.3 | 25 | **3.6** | **2.3** | **5.3** | 32 | **2.0** | **1.4** | **2.9** |
| Urinary bladder | 1 | 1.1 | 0.0 | 6.4 | 20 | 1.5 | 0.9 | 2.2 | 43 | 0.8 | 0.6 | 1.1 |
| Central nervous system | 2 | 3.3 | 0.4 | 12.0 | 6 | 1.9 | 0.7 | 4.2 | 7 | 1.2 | 0.5 | 2.6 |
| Hemo-lymphopoietic | 6 | 2.3 | 0.8 | 5.0 | 19 | 1.4 | 0.9 | 2.3 | 37 | 1.2 | 0.8 | 1.6 |
|  |  |  |  |  |  |  |  |  |  |  |  |  |

Obs= Observed cases. Statistical significant associations are highlighted in **bold.**

**^a^**Measured as Standardized incidence ratio (SIR), and 95% confidence intervals (CI). Men and women, age 0-84 years; second primary diagnosed <2 months after first one were excluded. **^b^**Poorly differentiated including anaplastic. **^c^**Cancer types with >30 cases in men and women.

**Appendix 4 (Table 4 Extended). Risk^a^ of thyroid cancers as second tumour by first cancer type, sex, and age. Italy, 1998-2012**

| **WOMEN** | **0-34 years** | | | | **35-54 years** | | | | **55-84 years** | | | |
| --- | --- | --- | --- | --- | --- | --- | --- | --- | --- | --- | --- | --- |
|  | **Obs** | **SIR** | **95 %** | **CI** | **Obs** | **SIR** | **95 %** | **CI** | **Obs** | **SIR** | **95 %** | **CI** |
| **TC after all neoplasms, but skin and TC** | 95 | **2.44** | **1.97** | **2.98** | 514 | **1.39** | **1.27** | **1.51** | 727 | **1.36** | **1.26** | **1.46** |
| Papillary TC, as second | 81 | **2.39** | **1.90** | **2.97** | 432 | **1.38** | **1.25** | **1.51** | 530 | **1.41** | **1.29** | **1.54** |
| Follicular TC, as second | 6 | 2.50 | 0.91 | 5.45 | 38 | **1.49** | **1.06** | **2.05** | 73 | **1.31** | **1.03** | **1.64** |
| Medullary TC, as second | 1 | 1.56 | 0.02 | 8.70 | 20 | **1.86** | **1.13** | **2.87** | 37 | **1.46** | **1.03** | **2.01** |
| Poorly differentiated **^b^** TC, as second | 1 | 16.18 | 0.21 | 90.03 | 5 | 1.78 | 0.57 | 4.15 | 35 | 1.30 | 0.90 | 1.80 |
|  |  |  |  |  |  |  |  |  |  |  |  |  |
| **Cancer types (first) ^c^** |  |  |  |  |  |  |  |  |  |  |  |  |
| Head and neck | 1 | 2.2 | 0.0 | 12.2 | 8 | 1.9 | 0.8 | 3.7 | 11 | 1.6 | 0.8 | 2.8 |
| Stomach | 0 | 0.0 | 0.0 | 10.4 | 4 | 0.8 | 0.2 | 2.0 | 14 | 1.0 | 0.5 | 1.6 |
| Colon and rectum | 3 | 2.6 | 0.5 | 7.6 | 37 | **1.5** | **1.1** | **2.1** | 95 | 1.2 | 1.0 | 1.5 |
| Lung | 0 | 0.0 | 0.0 | 13.0 | 14 | **3.2** | **1.8** | **5.4** | 15 | 1.3 | 0.7 | 2.1 |
| Melanoma | 9 | 1.5 | 0.7 | 2.9 | 29 | **1.6** | **1.0** | **2.2** | 22 | 1.5 | 0.9 | 2.2 |
| Breast | 13 | 1.4 | 0.8 | 2.5 | 255 | **1.2** | **1.1** | **1.4** | 315 | **1.4** | **1.2** | **1.5** |
| Corpus uteri | 0 | 0.0 | 0.0 | 6.6 | 27 | 1.3 | 0.9 | 1.9 | 60 | **1.4** | **1.1** | **1.8** |
| Cervix uteri | 2 | 0.7 | 0.1 | 2.7 | 19 | 1.2 | 0.7 | 1.9 | 11 | 1.3 | 0.7 | 2.4 |
| Ovary | 2 | 0.9 | 0.1 | 3.3 | 18 | 1.4 | 0.9 | 2.3 | 13 | 1.0 | 0.5 | 1.7 |
| Kidney and renal pelvis | 6 | **8.3** | **3.0** | **18.1** | 24 | **3.7** | **2.4** | **5.5** | 37 | **2.7** | **1.9** | **3.7** |
| Urinary bladder | 2 | 2.8 | 0.3 | 10.1 | 5 | 0.8 | 0.3 | 1.9 | 25 | 1.2 | 0.8 | 1.8 |
| Hemo-lymphopoietic | 34 | **3.7** | **2.6** | **5.2** | 44 | **1.9** | **1.4** | **2.6** | 50 | 1.3 | 1.0 | 1.7 |
|  |  |  |  |  |  |  |  |  |  |  |  |  |
| **MEN** |  |  |  |  |  |  |  |  |  |  |  |  |
| **TC after all neoplasms, but skin and TC** | 38 | **3.64** | **2.58** | **5.00** | 139 | **2.09** | **1.76** | **2.47** | 448 | **1.50** | **1.37** | **1.65** |
| Papillary TC, as second | 33 | **3.78** | **2.60** | **5.30** | 118 | **2.29** | **1.89** | **2.74** | 288 | **1.57** | **1.39** | **1.76** |
| Follicular TC, as second | 2 | 2.56 | 0.29 | 9.23 | 4 | 0.64 | 0.17 | 1.64 | 50 | 1.34 | 0.99 | 1.76 |
| Medullary TC, as second | 1 | 2.90 | 0.04 | 16.16 | 4 | 1.15 | 0.31 | 2.95 | 37 | **1.72** | **1.21** | **2.37** |
| Poorly differentiated **^b^** TC, as second | 0 | 0.00 | 0.00 | 98.87 | 3 | 2.33 | 0.47 | 6.82 | 30 | 1.44 | 0.97 | 2.05 |
|  |  |  |  |  |  |  |  |  |  |  |  |  |
| **Cancer types (first) ^c^** |  |  |  |  |  |  |  |  |  |  |  |  |
| Head and neck | 0 | 0.0 | 0.0 | 22.5 | 15 | **2.4** | **1.4** | **4.0** | 38 | **2.3** | **1.6** | **3.2** |
| Stomach | 0 | 0.0 | 0.0 | 33.1 | 0 | 0.0 | 0.0 | 1.4 | 12 | 1.2 | 0.6 | 2.1 |
| Colon and rectum | 0 | 0.0 | 0.0 | 10.7 | 20 | **2.2** | **1.4** | **3.4** | 75 | **1.6** | **1.2** | **2.0** |
| Lung | 1 | 9.6 | 0.1 | 53.3 | 6 | 2.0 | 0.7 | 4.5 | 30 | **1.7** | **1.1** | **2.4** |
| Melanoma | 4 | **3.9** | **1.0** | **9.9** | 23 | **4.5** | **2.9** | **6.8** | 20 | **2.9** | **1.8** | **4.4** |
| Prostate | 0 | 0.0 | 0.0 | 666.9 | 7 | 1.8 | 0.7 | 3.8 | 140 | **1.5** | **1.3** | **1.8** |
| Kidney and renal pelvis | 1 | 3.9 | 0.1 | 21.8 | 23 | **5.0** | **3.2** | **7.5** | 30 | **2.4** | **1.6** | **3.5** |
| Urinary bladder | 1 | 2.2 | 0.0 | 12.2 | 11 | 1.2 | 0.6 | 2.1 | 50 | 1.1 | 0.8 | 1.4 |
| Hemo-lymphopoietic | 18 | **5.9** | **3.5** | **9.4** | 16 | 1.6 | 0.9 | 2.6 | 21 | 1.0 | 0.6 | 1.6 |
|  |  |  |  |  |  |  |  |  |  |  |  |  |

Obs= Observed cases. Statistical significant associations are highlighted in bold.

^a^Measured as Standardized incidence ratio (SIR), and 95% confidence intervals (CI). Men and women, age 0-84 years; second primary cancers diagnosed <2 months after first one were excluded. ^b^Poorly differentiated including anaplastic. ^c^Cancer types with >30 cases in men and women.
